# Efficacy and safety of ublituximab for relapsing multiple sclerosis patients: current evidence and expert opinion

**DOI:** 10.1007/s00415-025-13442-0

**Published:** 2025-10-17

**Authors:** Paolo Preziosa, Vincenzo Brescia Morra, Marco Capobianco, Claudio Gasperini, Luigi M. E. Grimaldi, Girolama A. Marfia, Damiano Paolicelli, Paola Perini, Valentina Torri Clerici, Massimo Filippi

**Affiliations:** 1 Neuroimaging Research Unit, Division of Neuroscience, IRCCS San Raffaele Scientific Institute, Via Olgettina, 60, 20132 Milan, Italy; 2 Neurology Unit, IRCCS San Raffaele Scientific Institute, Via Olgettina, 60, 20132 Milan, Italy; 3 Neurorehabilitation Unit, IRCCS San Raffaele Scientific Institute, Via Olgettina, 60, 20132 Milan, Italy; 4 Neurophysiology Service, IRCCS San Raffaele Scientific Institute, Via Olgettina, 60, 20132 Milan, Italy; 5 Vita-Salute San Raffaele University, Milan, Italy; 6 Department of Neuroscience, Reproductive Science and Odontostomatology, Federico II University of Naples, Naples, Italy; 7 Multiple Sclerosis Unit, Policlinico Federico II University Hospital, Naples, Italy; 8 Neurology Department, “S. Croce e Carle” Hospital, Cuneo, Italy; 9 Department of Neurosciences, S Camillo Forlanini Hospital, Rome, Italy; 10 Neurology and Multiple Sclerosis Center, Fondazione Istituto “G. Giglio”, Cefalù, Italy; 11 Multiple Sclerosis Clinical and Research Unit, Fondazione Policlinico Tor Vergata, Department of Systems Medicine, University Tor Vergata, Rome, Italy; 12 Department of Translational Biomedicines and Neurosciences, University of Bari Aldo Moro, Bari, Italy; 13 Multiple Sclerosis Centre, Neurologic Clinic, University Hospital of Padova, Padua, Italy; 14 Neuroimmunology and Neuromuscular Diseases Unit, Fondazione IRCCS Istituto Neurologico Carlo Besta, Milan, Italy

**Keywords:** Multiple sclerosis, Ublituximab, Efficacy, Safety, Randomized controlled trial

## Abstract

Ublituximab, a newly launched anti-CD20 monoclonal antibody, represents a substantial advancement in the treatment landscape for relapsing multiple sclerosis (RMS). Its unique glycoengineered design enhances antibody-dependent cellular cytotoxicity, enabling rapid and effective B-cell depletion. Phase III randomized controlled trials ULTIMATE I and II confirmed superior efficacy of ublituximab over teriflunomide, achieving substantial reductions in annualized relapse rates, near-complete suppression of gadolinium-enhancing T1 lesions and new/enlarging T2-hyperintense white matter lesions, as well as higher rates of no evidence of disease activity 3. Safety data indicate that ublituximab is generally well tolerated, with mild, manageable infusion-related reactions as the most common adverse event. Its streamlined infusion protocol, requiring maintenance doses administered in just 1 h twice a year, provides a practical solution to the clinical and logistical challenges of MS management. Its rapid B-cell depletion, high efficacy, and convenient twice-yearly short infusion regimen make it particularly suitable for treatment-naïve patients with high disease activity who may benefit from early and robust disease control as well as for those who have experienced suboptimal responses, poor tolerability, or safety concerns with prior disease-modifying therapies. Although ublituximab shows great promise and five-year data are already available, further research is required to fully explore its potential in limiting disability progression and neurodegeneration, as well as to confirm its long-term safety. Real-world evidence, extended follow-ups, and comprehensive biomarker assessment specific to MS-related pathology will be essential to confirm its efficacy and optimize RMS patients’ management. This review synthesizes discussions from two meetings of Italian Neurologists held in 2024 and 2025, focusing on efficacy and safety data of ublituximab, and providing a comprehensive and in-depth analysis of its current and future role in RMS treatment.

## Introduction

Multiple sclerosis (MS) is a chronic, inflammatory, demyelinating, and neurodegenerative disease of the central nervous system (CNS), characterized by a progressive accumulation of clinical disability over time [1]. In recent years, the treatment landscape for MS has expanded significantly, with more than 20 disease-modifying therapies (DMTs) now available [2]. Among these, anti-CD20 monoclonal antibodies (mAbs) have emerged as a cornerstone of effective MS management due to their strong efficacy in reducing relapses, suppressing magnetic resonance imaging (MRI) activity, and contributing to delaying disability progression [3–7].

Anti-CD20 therapies deplete B-cells, targeting their pathogenic role in MS immune pathophysiology [3, 4, 6, 8–11]. Currently, four anti-CD20 mAbs are available for MS treatment: rituximab (off-label), ocrelizumab, ofatumumab, and the most recently approved agent, ublituximab. Ublituximab, a glycoengineered chimeric mAb designed to enhance antibody-dependent cellular cytotoxicity (ADCC), was approved by the Food and Drug Administration (FDA) in December 2022 and by the European Medicines Agency (EMA) in May 2023. Ublituximab is currently indicated for adults with relapsing MS (RMS). In the USA [12], this includes clinically isolated syndrome (CIS), relapsing–remitting MS (RRMS), and active secondary progressive MS (SPMS) [13]. In the European Union [14], it is approved for adults with RMS with active disease defined by clinical or imaging features [13]. In pivotal phase III randomized controlled trials (RCTs) ULTIMATE I and II [15], ublituximab showed superior efficacy in reducing annualized relapse rates (ARR) and MRI lesion burden compared to teriflunomide, further solidifying its role as a highly effective disease-modifying treatment.

As new data on ublituximab continue to emerge, a comprehensive update on its efficacy and safety is crucial. This is particularly relevant for ublituximab, given its unique properties, its recent introduction into clinical practice in some countries, and its anticipated availability in others. A deeper understanding of ublituximab’s efficacy and safety can guide personalized treatment strategies for RMS patients, ensuring optimal therapeutic outcomes while minimizing safety and tolerability risks.

To provide an updated perspective on ublituximab, two meetings of Italian Neurologists with expertise in MS were convened on September 24, 2024, and January 15, 2025. These meetings aimed to consolidate the latest knowledge on efficacy and safety of ublituximab in RMS. Key topics included B-cell depletion mechanisms, clinical and MRI outcomes, safety considerations—including adverse events (AEs), infusion-related reactions (IRRs), and immunogenicity—and the identification of MS patients most suitable for this therapy (see Table 1 for research strategy and section criteria). Main findings and expert opinions were synthesized into a draft document, which underwent critical review and revision also by additional specialists. This review aims to summarize the discussions and findings from these expert meetings, providing clinicians with a comprehensive evaluation of therapeutic potential of ublituximab in RMS management.

**Table 1:** Search strategy and selection criteria

Sources	References for this Review were identified through searches of Pubmed (https://www.ncbi.nlm.nih.gov/pubmed) Articles were also identified through searches of the authors’ own files. Abstracts presented at main congresses in the field were also evaluated
Period of time covered	Correct reporting from January 1, 1979, to January 31, 2025
Search terms	References for this Review were identified through searches of PubMed (https://www.ncbi.nlm.nih.gov/pubmed) with the following search terms: ‘activity,’ ‘adverse event,’ ‘antibody dependent cellular cytotoxicity,’ ‘anti-drug antibodies,’ ‘antigen-presenting cell,’ ‘atrophy,’ ‘B-cell,’ ‘CD19,’ ‘CD20,’ ‘cognition,’ ‘depleting therapy(ies),’ ‘disability,’ ‘disability improvement,’ ‘disability progression,’ ‘disease-modifying,’ ‘efficacy,’ ‘gadolinium,’ ‘hypogammaglobulinemia,’ ‘immunoglobulin,’ ‘immunology,’ ‘immunogenicity,’ ‘immuno-related reaction,’ ‘immunosuppression,’ ‘infection,’ ‘infusion,’ ‘lesions,’ ‘leukopenia,’ ‘long-term,’ ‘lymphocyte,’ ‘lymphopenia,’ ‘open-label extension,’ ‘mechanism of action,’ ‘magnetic resonance imaging,’ ‘monoclonal antibody(ies),’ ‘multiple sclerosis,’ ‘neoplasm,’ ‘neutralizing antibodies,’ ‘no evidence of disease activity,’ ‘outcome,’ ‘pathology,’ ‘phase II,’ ‘phase III,’ ‘phenotype(s),’ ‘randomized controlled trial,’ ‘relapsing,’ ‘relapsing–remitting,’ ‘safety,’ ‘secondary progressive,’ ‘serious adverse event,’ ‘tolerability,’ ‘ublituximab,’ ‘Ultimate,’ ‘volume loss,’ ‘white matter.’
Selection criteria and review preparation	1. Only papers published in English 2. The final reference list was generated with the consensus of all co-authors of this review on the basis of originality and relevance to the broad scope of this review, with a focus on the most recent articles published in the last five years 3. Experts provided a summary during the meetings of the main findings related to specific topics of the review. For each topic, a group consensus was reached and summarized in a first draft, which was circulated among the co-authors for further critical discussion and revision. The review represents the final conclusions reached by co-authors

## The rationale of anti-CD20 therapies in MS

The advent of anti-CD20 therapies has revolutionized MS management by targeting a key driver of the disease. The rationale for anti-CD20 therapies in MS is rooted in the critical role of B-cells in MS pathophysiology [3, 4, 6, 8–11]. Historically, MS was considered a T cell-mediated disease; however, increasing evidence has consistently shown that B-cells also contribute significantly to disease activity and progression [3, 4, 6, 8, 9, 11]. B-cells play multiple roles in MS pathology, acting as antigen-presenting cells, secreting pro-inflammatory cytokines (e.g., interleukin-6 and granulocyte–macrophage colony-stimulating factor), and forming CNS-localized immune aggregates resembling ectopic lymphoid tissues [3, 4, 6, 8]. Pathological studies confirm an abundance of CD20+ B-cells in active MS lesions, especially in early and relapsing stages of the disease [1, 8, 16, 17]. Moreover, lymphoid aggregates are associated with subpial cortical demyelination, neuronal loss, and chronic inflammation [1, 8, 16, 18].

Anti-CD20 mAbs, including rituximab, ocrelizumab, ofatumumab, and the recently approved ublituximab, target CD20, a transmembrane protein expressed on B-cells from the pre-B-cell stage to memory B-cells [3–6, 9, 19, 20]. These therapies deplete B-cells via mechanisms such as antibody-dependent cellular cytotoxicity (ADCC), complement-dependent cytotoxicity (CDC), and direct apoptosis [3–6, 9, 19, 20]. By reducing the population of pathogenic B-cells, anti-CD20 mAbs decrease pro-inflammatory cytokine production, lower autoreactive T-cell activation, and suppress the formation of new inflammatory lesions [3–6, 9, 19, 20]. The efficacy of anti-CD20 therapies in MS is evident from their significant impact on reducing clinical relapses, suppressing MRI lesion activity (e.g., new T2-hyperintense white matter [WM] lesions and gadolinium [Gd]-enhancing T1 lesions), slowing disability progression, and limiting irreversible tissue loss in RRMS and, for ocrelizumab, also in primary progressive MS [3–6, 9, 19–21]. However, B-cell depletion can also increase the risk of adverse effects, such as hypogammaglobulinemia and serious infections, which must be carefully monitored during treatment [3].

## Pharmacology of ublituximab

**Fig. 1:**
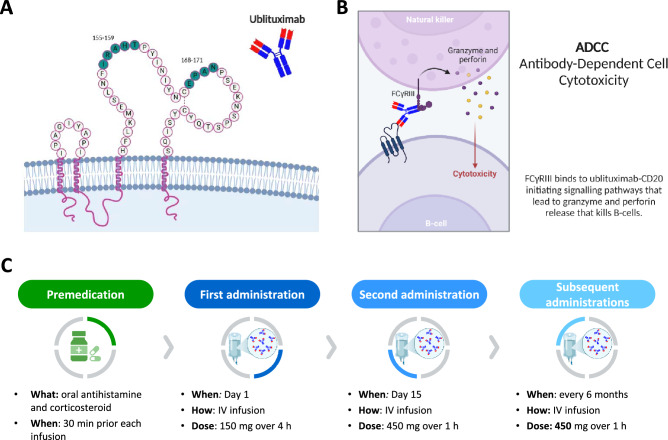
**A** Ublituximab target epitopes. Ublituximab binds to residues 155–159 and 168–171 on the large extracellular loop of CD20. **B** Main mechanism of action of ublituximab. Antibody-dependent cellular cytotoxicity (ADCC) is the most relevant effector mechanism of ublituximab [9]. This is primarily due to the glycoengineered Fc region, which has been afucosylated to enhance FcγRIIIa binding and recruitment of immune effector cells [51]. **C** Route of administration and dosing schedule of ublituximab. See text for further details. Created in https://BioRender.com

Ublituximab is a chimeric IgG1 kappa mAb designed to specifically target the large extracellular loop of CD20 at amino acid residues 155–159 and 168–171 (Fig. 1A) [3–5, 22, 23]. It features a glycoengineered fragment crystallizable (Fc) segment, optimized to enhance binding affinity for FCγRIIIa receptors, thereby preferentially promoting ADCC over CDC (Fig. 1B) [3–5, 9, 23]. However, the clinical implications of this enhanced ADCC compared with other approved anti-CD20 monoclonal antibodies, such as ocrelizumab or ofatumumab, have not yet been fully demonstrated in head-to-head studies and should therefore be interpreted cautiously.

Ublituximab is administered via intravenous infusion, starting with an initial dose of 150 mg infused over 4 h on Day 1, followed by a 450 mg dose administered over 1 h on Day 15 (Fig. 1C) [3, 4, 12, 14, 23]. Subsequent maintenance doses of 450 mg are given over 1 h every 24 weeks from Week 24 onwards [3, 4, 12, 14, 23]. Premedication includes oral or IV antihistamines and corticosteroids, which are administered 30–60 min before each infusion to mitigate IRRs.

Across both Phase II and Phase III ULTIMATE I and II RCTs, ublituximab treatment showed rapid depletion of CD19+ B-cells, with reductions of approximately > 95% observed as early as 24 h after the first infusion [15, 22]. The median time to B-cell repletion, defined as return to either the lower limit of normal (LLN) or baseline levels, was 70.3 weeks following the final infusion.

## Efficacy of ublituximab

### Phase II RCT

#### Study design

A 48-week, multicenter, placebo-controlled Phase II RCT evaluated the efficacy and safety of ublituximab in adults with RMS [22]. Participants met the 2010 McDonald criteria for RMS diagnosis and had Expanded Disability Status Scale (EDSS) scores between 0 and 5.5. Eligibility required neurological stability for at least 30 days before screening, two or more relapses in the previous two years or at least one relapse within one year prior to screening, and/or at least one Gd-enhancing lesion on brain MRI. A total of 48 RMS patients were randomized in a 3:1 ratio to receive either ublituximab or placebo across six dosing cohorts, designed to determine the optimal infusion protocol for safety and efficacy. The dosing regimen included an initial infusion of 150 mg ublituximab (1–4 h), followed by 450 mg or 600 mg on Day 15 (administered over 1–3 h) and at Week 24 (administered over 1–1.5 h) [4, 5, 7, 12, 14, 22–24]. Of the 48 RMS patients, 45 (94%) completed the study. Reasons for early withdrawal were one pregnancy (resulting in the delivery of a healthy infant), one relocation, and the departure of a treating investigator (one participant).

#### Primary endpoint B-cell depletion

The primary endpoint was B-cell depletion, defined as ≥ 95% reduction in CD19+ B-cells from baseline by Week 4 [22]. Ublituximab achieved strong B-cell depletion, with a median reduction exceeding 99% by Week 4 across all RMS patients, which was sustained throughout the 48-week study period.

#### Secondary endpoints

The mean ARR decreased by 95%, from 1.45 at baseline to 0.07, with 93% of RMS patients remaining relapse-free during the study [22]. At Week 48, ublituximab also showed promising effects on disability status, with 24-week confirmed disability improvement (CDI) observed in 17% of RMS patients (*n* = 8), whereas only 8% (*n* = 4) experienced 24-week confirmed disability progression (CDP).

Ublituximab led to almost complete suppression of MRI activity. No Gd-enhancing lesion was detected at Weeks 24 and 48 (100% reduction from baseline, *p* = 0.003). Brain T2-hyperintense WM lesion volume decreased by 10.6% at Week 48 (*p* = 0.002). Between baseline and Week 24, seven RMS patients (15%) developed eight new/enlarging T2-hyperintense WM lesions (mean = 0.20, standard deviation [SD] = 0.43), whereas only one RMS patient developed two new/enlarging brain T2-hyperintense WM lesions between Week 24 and Week 48 (mean = 0.04, SD = 0.29). Overall, by Week 48, 74% of evaluable RMS patients (34/76) achieved no evidence of disease activity 3 (NEDA-3) status, defined as no relapses, no MRI disease activity, and no CDP. However, given the very small sample size of this study (*n* = 48), these findings provided only preliminary evidence, particularly for ARR and MRI outcomes.

### Phase III RCTs

#### Study design

The ULTIMATE I (NCT03277261) and ULTIMATE II (NCT03277248) were double-blind, double-dummy phase III RCTs, comparing intravenous ublituximab to oral teriflunomide in RMS patients aged 18–55 years with EDSS scores of 0–5.5 and at least two relapses in the previous 2 years, or one relapse or at least one gadolinium-enhancing T1 lesion or both in the year before screening [15].

RMS patients were randomized 1:1 to receive either ublituximab (150 mg on Day 1, 450 mg on Day 15, and every 24 Weeks thereafter) or oral teriflunomide (14 mg daily). A total of 274 and 272 RMS patients received ublituximab in ULTIMATE I and II, respectively, whereas 275 and 273 RMS patients received teriflunomide.

#### B-cell depletion

Ublituximab achieved a 96% reduction in median CD19+ B-cell counts within 24 h of the first dose [15]. By the end of the double-blind period, CD19+ B-cell counts were reduced by 97%.

#### Clinical outcomes

**Fig. 2:**
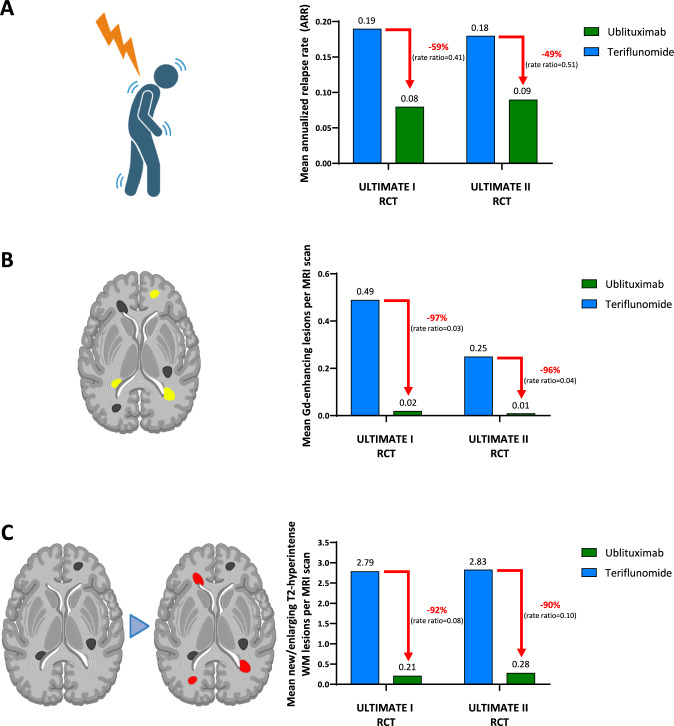
Primary endpoint of efficacy and MRI endpoints of disease activity of ULTIMATE I and II randomized controlled trials (RCTs). **A** The primary endpoint was annualized relapse rate (ARR). Mean number of **B** gadolinium (Gd)-enhancing T1 lesions and **C** new/enlarging T2-hyperintense WM lesions per MRI scan. Created in https://BioRender.com

Both RCTs met the primary outcome, showing significantly lower ARR in the ublituximab group compared to the teriflunomide group. In ULTIMATE I, the ARR was 0.08 with ublituximab versus 0.19 with teriflunomide, corresponding to a 59% relative reduction (rate ratio = 0.41, 95% confidence interval [CI] = 0.27; 0.62, *p* < 0.001) [12, 14]. In ULTIMATE II, the ARR was 0.09 with ublituximab versus 0.18 with teriflunomide, reflecting a 49% relative reduction (rate ratio = 0.51, 95% CI = 0.33; 0.78, *p* = 0.002) (Fig. 2A). However, these clinical benefits did not extend to disability progression outcomes. Pooled analysis showed comparable rates of 12-week CDP (5.2% versus 5.9%; hazard ratio [HR] = 0.84, 95% CI = 0.50; 1.41, *p* = 0.51) and 24-week CDP (3.3% versus 4.8%; HR = 0.66, 95% CI = 0.36; 1.21). However, ublituximab led to higher rates of 12-week (12.0% versus 6.0%, HR = 2.16, 95% CI = 1.41; 3.31) and 24-week CDI (9.6% versus 5.1%, HR = 2.03, 95% CI = 1.27; 3.25).

Ublituximab was associated with greater improvements in Multiple Sclerosis Functional Composite (MSFC) scores at Week 96: In ULTIMATE I, the mean improvement was 0.47 points versus 0.27 points in ULTIMATE I, and 0.52 points versus 0.28 points in ULTIMATE II. Conversely, the percentage of RMS patients with worsening performance on the Symbol Digit Modalities Test (SDMT) (≥4-point decrease from baseline at any post-baseline assessment up to Week 96) was comparable between the two treatment groups (ULTIMATE I: 29.2% for ublituximab and 31.8% for teriflunomide; ULTIMATE II: 29.0% for ublituximab and 31.6% for teriflunomide).

#### MRI outcomes

When compared to teriflunomide, ublituximab resulted in a 97% relative reduction in average number of Gd-enhancing T1 lesions per MRI scan in the ULTIMATE I trial (0.02 versus 0.49, rate ratio = 0.03, 95% CI = 0.02; 0.06, *p* < 0.001) and 96% relative reduction in the ULTIMATE II trial (0.01 versus 0.25, rate ratio = 0.04, 95% CI = 0.02; 0.06, *p* < 0.001) (Fig. 2B) [15]. Similarly, the number of new/enlarging T2-hyperintense WM lesions was reduced by 92% in ULTIMATE I (0.21 versus 2.79, rate ratio = 0.08, 95% CI = 0.06; 0.10, *p* < 0.001) and 90% in ULTIMATE II (0.28 versus 2.83, rate ratio = 0.10, 95% CI = 0.07; 0.14, *p* < 0.001) (Fig. 2C). However, no significant differences were observed in brain volume changes between treatment groups in either RCTs (ULTIMATE I: least squares mean change = − 0.20 for ublituximab versus -0.13 for teriflunomide, mean difference = − 0.07, 95% CI = − 0.11; − 0.04; ULTIMATE II: least squares mean change = − 0.19 versus − 0.18, respectively, mean difference = − 0.02, 95% CI = − 0.05; 0.02), highlighting the need for cautious interpretation of efficacy beyond relapse and lesion control at least during the 2-year time frame of RCTs.

#### NEDA-3

**Fig. 3:**
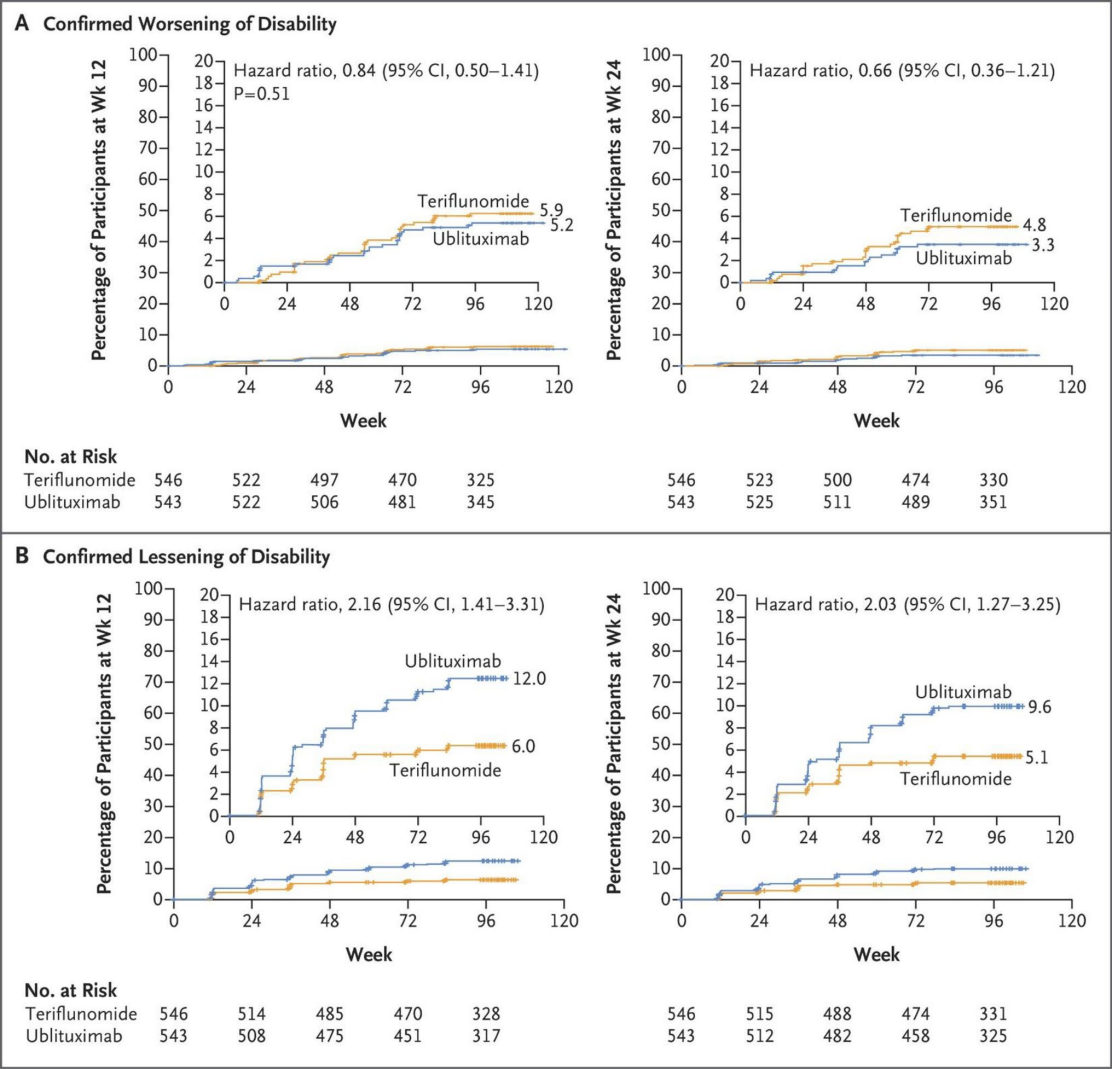
Confirmed worsening of disability and lessening of disability of ULTIMATE I and II RCTs. Shown are Kaplan–Meier estimates of the percentages of participants in the modified intention-to-treat population with worsening of disability confirmed at 12 weeks and at 24 weeks (**A**) and of participants with lessening of disability confirmed at 12 weeks and at 24 weeks (**B**) pooled across both trials. The modified intention-to-treat population included all participants who received at least one dose of a trial drug and had one baseline and at least one post-baseline efficacy assessment. Because of the failure in the hierarchical testing of the end point of worsening of disability confirmed at 12 weeks, between-group differences in worsening of disability at 24 weeks and lessening of disability were not considered to be significant. Participants were at risk until Week 84; worsening of disability or lessening of disability that first occurred at Week 96 could not be confirmed. Hazard ratios were estimated with the use of a Cox regression model with treatment group as a covariate. The tick marks indicate censored data. The inset in each panel shows the same data on an enlarged *y* axis. From [15] with permission

In the ULTIMATE I trial, NEDA-3 was achieved in 44.6% of RMS patients in the ublituximab group compared to 15.0% in the teriflunomide group (odds ratio [OR] = 5.44, 95% CI = 3.54; 8.38). Similar findings were reported in the ULTIMATE II trial, with NEDA rates of 43.0% and 11.4%, respectively (OR = 7.95, 95% CI = 4.92; 12.84) (Fig. 3).

### Post hoc analyses of phase III RCTs

Following the phase III RCTs, various post hoc analyses were conducted, offering deeper insights into the efficacy of ublituximab. However, because these analyses were not pre-specified, their results should be interpreted cautiously due to the elevated risk of false positive findings. Moreover, multiple comparisons across numerous endpoints and subgroups may inflate statistical significance despite adjustments, further warranting cautious interpretation. Finally, several sources cited in this section are conference abstracts or posters and should be regarded as preliminary until full peer-reviewed publications are available.

#### NEDA-3 and NEDA-4

Pooled post hoc analyses of the ULTIMATE I and II Phase III RCTs further explored the efficacy of ublituximab versus teriflunomide in achieving NEDA-3 [25]. These analyses examined treatment effects across different periods, including Weeks 0–96, Weeks 24–96, and Weeks 48–96 (re-baselined), as well as in subgroups defined by age (≤ 38 or > 38 years), disease duration (< 3 or ≥ 3 years since diagnosis), treatment history (treatment-naïve or prior DMT use), presence of Gd-enhancing lesions (0 or ≥ 1), and EDSS score (≤ 3.5 or > 3.5).

Ublituximab significantly outperformed teriflunomide in achieving NEDA-3 during the entire 96-week period and in re-baselined intervals [25]. Over Weeks 0–96, 44.6% of RMS patients treated with ublituximab achieved NEDA-3 compared to 12.4% for teriflunomide, representing a 3.6-fold improvement (OR = 7.36, 95% CI = 5.30; 10.23, *p* < 0.0001). When re-baselined from Week 24, NEDA-3 was achieved by 82.1% of ublituximab-treated patients versus 22.5% in the teriflunomide group (OR = 17.94, 95% CI = 12.93; 24.89, *p* < 0.0001). Similarly, during Weeks 48–96, 88.2% of RMS patients treated with ublituximab achieved NEDA-3 compared to 30.4% of those receiving teriflunomide (OR = 19.66, 95% CI = 13.74; 28.11, *p* < 0.0001). Over the entire 96-week period, new/enlarging T2-hyperintense WM lesions were the main driver of NEDA-3 failure in both treatment groups, occurring in 44.8% of RMS patients in the ublituximab group and 81.1% of those receiving teriflunomide. However, in the re-baselined intervals (Weeks 24–96 and Weeks 48–96), the contributors to disease activity diverged. Among teriflunomide-treated RMS patients, new/enlarging T2-hyperintense WM lesions remained the predominant driver, occurring in 71.6% and 63.4% of RMS patients, respectively. Conversely, among ublituximab-treated RMS patients, relapses became the primary contributor, although they were observed in only 11.4% of RMS patients during Weeks 24–96 and 7.6% during Weeks 48–96. Across all evaluated subgroups, ublituximab consistently showed superior efficacy over teriflunomide in achieving NEDA-3, with the greatest benefits observed in younger patients, those with shorter disease duration, lower EDSS scores, and the presence of Gd-enhancing lesions [25].

A separate post hoc analysis evaluated NEDA-4, which incorporates the absence of excessive brain atrophy between Weeks 24 and 96 [26]. Brain volume loss was assessed using the Jacobian integration method and defined at annual thresholds of 0.4%, 0.8%, and 1.2% [26]. Across all thresholds, significantly greater proportions of RMS patients treated with ublituximab achieved NEDA-4 compared to those receiving teriflunomide (at 0.4% threshold: 44.2% versus 13.5%, OR = 5.48 [95% CI = 4.03; 7.46, *p* < 0.0001; at 0.8% threshold: 68.0% versus 19.0%, OR = 10.10 [95% CI = 7.50; 13.60, *p* < 0.0001; at 1.2% threshold: 71.9% versus 19.6%, OR = 11.69 (95% CI = 8.64; 15.81, *p* < 0.0001) [26]. Across all evaluated thresholds of annual brain volume loss, a smaller proportion of ublituximab-treated RMS patients exceeded the defined limits compared to those receiving teriflunomide (at 0.4% threshold: 49.5 versus 54.0%; at 0.8% threshold: 21.2% versus 25.8%; at 1.2% threshold: 16.1% versus 18.8%). Even though these NEDA-4 results rely on brain atrophy thresholds that are not standardized regulatory endpoints and therefore should be interpreted with particular caution, the concordance of findings across different thresholds supports the superiority of ublituximab over teriflunomide in achieving this composite outcome.

Interestingly, among RMS patients who did not achieve NEDA-4, brain volume loss was the predominant driver of NEDA-4 failure in the ublituximab group, whereas new/enlarging T2-hyperintense WM lesions were the primary contributor in the teriflunomide group.

#### Efficacy in highly active RMS patients

A pooled post hoc analysis evaluated ublituximab in RMS patients with highly active disease, defined as those with at least two relapses in the previous year and at least one Gd-enhancing lesion at baseline (ublituximab *n* = 88, teriflunomide *n* = 80) [27]. At Week 96, ublituximab showed a 70.8% relative reduction in ARR compared to teriflunomide (0.145 versus 0.496, *p* < 0.001), a 95.7% decrease in Gd-enhancing T1 lesions per MRI scan (0.038 versus 0.875, *p* < 0.001), and a 91.1% reduction in new/enlarging T2-hyperintense WM lesions per MRI scan (0.568 versus 6.367, *p* < 0.001). Ublituximab-treated RMS patients were 4.8 times more likely to achieve NEDA-3 during Weeks 24–96 compared to those receiving teriflunomide (77.9% versus 16.4%). Percent brain volume change (− 0.614% for ublituximab versus − 0.664% for teriflunomide) and proportion of 12-week CDP (8.0% for ublituximab, 5.0% for teriflunomide) were comparable between groups.

Another post hoc analysis conducted at Week 12 in a subgroup of highly active RMS patients evaluated the rapid onset of ublituximab’s efficacy [28]. By Week 12, ublituximab reduced the number of Gd-enhancing T1 lesions by 83% compared to teriflunomide (least squares mean: 0.114 versus 0.683, *p* < 0.0001), with 88% of highly active RMS patients achieving freedom from Gd-enhancing lesions. Ublituximab also significantly reduced the number of new/enlarging T2-hyperintense WM lesions by 58% (least squares mean: 1.754 versus 4.127, *p* < 0.0001). Additionally, significantly more RMS patients treated with ublituximab achieved NEDA-3 at Week 12 compared to those receiving teriflunomide (30% versus 10%, *p* = 0.0012).

#### Efficacy in naïve RMS patients

Two other post hoc analyses specifically examined the efficacy of ublituximab in treatment-naïve RMS patients [25, 29]. In the first analysis, which included 345 RMS patients treated with ublituximab and 377 with teriflunomide, ublituximab was associated with a significant 56.9% relative reduction in the ARR (0.081 versus 0.188, *p* < 0.001), a 96.1% reduction in the number of Gd-enhancing lesions per MRI scan (0.031 versus 0.791, *p* < 0.001), and a 90.6% reduction in new/enlarging T2-hyperintense WM lesions (0.390 versus 4.144, *p* < 0.001) [29]. Additionally, a significantly higher proportion of ublituximab-treated patients achieved NEDA-3 status at Week 96 compared to Week 24 (82.7% versus 23.1%, *p* < 0.001) [29]. Ublituximab was also associated with a significantly higher rate of 12-week CDI compared to teriflunomide (11.2% versus 5.5%, *p* = 0.0095) and greater improvements in MSFC scores from baseline (0.53 versus 0.28, *p* = 0.0047).

Further subgroup analyses showed that improvements in EDSS and MSFC scores were particularly pronounced in patients treated within 3 years of symptom onset, with greater benefits observed in this subgroup compared to those with a longer disease duration [25]. Among treatment-naïve RMS patients whose first MS symptom occurred within three years prior to enrollment, ublituximab showed a significantly greater improvement in EDSS scores compared to teriflunomide (− 0.16 versus 0.02, *p* = 0.0086). In RMS patients with a longer disease duration, ublituximab showed a numerical improvement in EDSS scores (− 0.10 versus 0.01), although this did not reach statistical significance (*p* = 0.0511). Similarly, changes in MSFC scores favored ublituximab, with more pronounced benefits in patients treated earlier in their disease course (≤ 3 years: 0.32 versus 0.09, a 3.6-fold improvement; > 3 years: 0.63 versus 0.36, a 1.8-fold improvement).

#### Effects on thalamic atrophy

A post hoc analysis of thalamic volume changes over 96 weeks in 537 RMS patients per treatment group showed that ublituximab promoted a 22% relative reduction of thalamic atrophy compared to teriflunomide (percentage change: − 1.34% versus − 1.71%, *p* = 0.0013) [30]. Although statistically significant, the absolute difference is limited, and the clinical relevance of this magnitude of thalamic volume change needs to be better explored.

#### Other post hoc analyses

A separate post hoc analysis assessed the impact of ublituximab on EDSS scores and its individual Functional Systems Scores (FSS) [31]. Ublituximab significantly improved EDSS scores across all visits, with an OR of 1.7 (95% CI = 1.25; 2.37, *p* = 0.0010). Significant improvements were also observed in sensory function (OR = 1.4, 95% CI = 1.12; 1.88, *p* = 0.0052) and bowel/bladder functions (OR = 1.4, 95% CI = 1.05; 1.81, *p* = 0.0222). Significant improvements in EDSS and sensory function were particularly notable between Weeks 48 and 96, while bowel and bladder function improvements were observed as early as Week 24 and remained significant through Week 96. Additional functional benefits were seen in cerebellar function at Weeks 48, 84, and 96, cerebral/mental function at Weeks 48, 72, and 84, and pyramidal function and ambulation at Week 96.

Another post hoc analysis evaluated disability progression in a subset of RMS patients who remained relapse-free (ublituximab *n* = 473, teriflunomide *n* = 406) [32]. Compared to teriflunomide, ublituximab led to significant improvements in EDSS scores at multiple timepoints, including Week 48 (− 0.13 versus − 0.06), Week 84 (− 0.19 versus − 0.08), and Week 96 (− 0.19 versus − 0.07) (*p* < 0.05 for all comparisons). RMS patients treated with ublituximab also exhibited better outcomes in MSFC scores (least squares mean change from baseline = 0.557 versus 0.359, *p* = 0.0095), 9-Hole Peg Test (least squares mean change = 0.152 versus 0.025, *p* = 0.0005) and the Timed 25-Foot Walk test (least squares mean change = 0.071 versus − 0.025, *p* = 0.0375) from baseline to Week 96.

A further post hoc analysis assessed disease activity using a serum-based Multiple Sclerosis Disease Activity (MSDA) test, which quantifies 18 biomarkers and generates a Disease Activity (DA) score [33]. Ublituximab significantly reduced DA scores, with 100% of RMS patients achieving low DA scores by Week 96, compared to 30% in the teriflunomide group (*p* < 0.05 after Bonferroni correction). Additionally, ten biomarkers (NEFL, MOG, CXCL13, OPG, TNFRSF10A, PRTG, FLRT2, TNFSF13B, OPN, and GFAP) showed significant reductions in the ublituximab group compared to teriflunomide. Comparable area under the receiver operating characteristic curve values supported ublituximab’s efficacy in managing disease activity, highlighting the potential of the MSDA test as a quantitative tool for assessing disease activity and therapeutic response. However, these MSDA findings are exploratory and should be considered hypothesis-generating rather than confirmatory.

## Safety and tolerability of ublituximab

### Adverse events and serious adverse events

In the phase II RCT, ublituximab was generally well tolerated [22]. No patient discontinued the study due to a drug-related adverse event (AE). Only one grade 3 AE (fatigue) was considered possibly related to ublituximab. No serious infections were reported, and no deaths occurred during the study. The most frequently reported grade 1 or 2 AEs were IRRs (58%), arthralgia (15%), nausea (15%), and upper respiratory tract infection (15%). IRRs were most common on the day of the first infusion.

In the pooled analysis of the ULTIMATE I and II RCTs, 486 of 545 RMS patients (89.2%) who received ublituximab and 501 of 548 RMS patients (91.4%) who received teriflunomide experienced at least one AE (Table 2) [15]. Grade 3 or higher AEs were observed in 116 RMS patients (21.3%) in the ublituximab group and in 77 (14.1%) in the teriflunomide group [15]. The most frequently observed AEs included IRRs, headache, nasopharyngitis, pyrexia, and nausea (Table 2). Compared with teriflunomide, IRRs were markedly more common (47.7% vs 12.2%), and both serious adverse events (SAEs) and adverse event (AE)-related treatment discontinuations occurred more frequently in the ublituximab group (SAEs: 10.8% vs 7.3%; discontinuations: 4.2% vs 0.7%) (Table 2).

**Table 2:** Adverse events in the pooled safety population of ULTIMATE I and II RCTs

AE	Pooled ULTIMATE I and II RCTs	
	Ublituximab (*n* = 545)	Teriflunomide (*n* = 548)
Any AE	486 (89.2%)	501 (91.4%)
AE leading to treatment discontinuation	23 (4.2%)	4 (0.7%)
Infusion-related reaction	260 (47.7%)	67 (12.2%)
Blood and lymphatic system disorders	81 (14.9%)	70 (12.8%)
Leukopenia	11 (2.0%)	19 (3.5%)
Lymphopenia	53 (9.7%)	6 (1.1%)
Neutropenia	18 (3.3%)	23 (4.2%)
Gastrointestinal disorders	163 (29.9%)	174 (31.8%)
Nausea	58 (10.6%)	43 (7.8%)
Headache	187 (34.3%)	146 (26.6%)
Pyrexia	76 (13.9%)	27 (4.9%)
Infection	304 (55.8%)	298 (54.4%)
Nasopharyngitis	100 (18.3%)	98 (17.9%)
Respiratory tract infection	42 (7.7%)	38 (6.9%)
Upper respiratory tract infection	41 (7.5%)	38 (6.9%)
Pharyngitis	32 (5.9%)	12 (2.2%)
Urinary tract infection	22 (4.0%)	29 (5.3%)
Neoplasm*	2 (0.4%)	1 (0.2%)
SAE^^^	59 (10.8%)	40 (7.3%)
Serious infection	27 (5.0%)	16 (2.9%)
Death^#^	3 (0.6%)	0 (0.0%)

*AE* adverse event, *RCT* randomized controlled trial, *SAE* serious adverse event

*In both RCTs, neoplasms that occurred in the ublituximab group were endometrial (time to onset, 558 days) and uterine (time to onset, 210 days). A tongue neoplasm (time to onset, 494 days) occurred in the teriflunomide group

^^^In both RCTs, the most frequently reported serious infections were pneumonia in the ublituximab group and urinary tract infections in the teriflunomide group

^#^The deaths that occurred in the ublituximab group were due to pneumonia (deemed to be possibly related to treatment), encephalitis (after measles), and salpingitis (after ectopic pregnancy)

Among the ublituximab-treated group, notable SAEs included pneumonia, encephalitis after measles, and salpingitis after ectopic pregnancy, each accounting for one death. No deaths occurred in the teriflunomide group. Treatment discontinuation due to AEs was observed in 4.2% of the ublituximab group and 0.7% of the teriflunomide group.

### Infusion-related reactions

In the Phase II RCT, IRRs were reported in 24 RMS patients (50%); however, of the total 141 total ublituximab infusions, 77% did not result in an IRR [22]. All IRRs were classified as Grade 1 or 2 in severity and primarily occurred on the first infusion day (*n* = 21; 44%) [22]. The frequency of IRRs on Day 15 and at Week 24 did not appear to increase with higher doses or faster infusion times.

In the pooled analysis of the ULTIMATE I and II RCTs, IRRs were reported in 47.7% of RMS patients who received ublituximab. The most frequently reported IRR-related symptoms included pyrexia, headache, chills, and influenza-like illness. Most IRRs were mild to moderate in severity, and the majority occurred during the first infusion (43.3%). The frequency of IRRs decreased with subsequent doses. Grade 3 or higher IRRs were observed in 2.8% of the RMS patients receiving ublituximab. Two RMS patients experienced grade 4 IRRs: one developed anaphylaxis during the second infusion and recovered following treatment but did not receive further doses, while the other experienced a lymphocyte count decrease during the first infusion, which did not require treatment or dose modification. A total of six RMS patients (1.1%) discontinued ublituximab due to IRRs, with three discontinuations occurring during the first infusion and three after the first infusion.

### Infections

No serious infections or were reported in the Phase II RCT [22].

In the pooled analysis of the ULTIMATE I and II RCTs, infections occurred in 304 RMS patients (55.8%) who received ublituximab and in 298 RMS patients (54.4%) who received teriflunomide [15]. Most infections were related to the respiratory tract and were classified as Grade 1 or 2 in severity. Nasopharyngitis occurred in 18.3% of RMS patients in the ublituximab group and in 17.9% of teriflunomide group. Respiratory tract infections occurred in 7.7% and 6.9%, respectively, whereas pharyngitis occurred in 5.9% and 2.2%, respectively. The incidence of urinary tract infections was comparable between groups (4.0% vs. 5.3%). Serious infections occurred in 5.0% of RMS patients in the ublituximab group and in 2.9% of teriflunomide group, thus involving a minority of patients but underscoring the need for vigilant infection risk assessment and monitoring during therapy. The most frequently reported serious infection in the ublituximab group was pneumonia, whereas urinary tract infections were the most common serious infections in the teriflunomide group. Seven RMS patients (1.3%) receiving ublituximab and one (0.2%) receiving teriflunomide discontinued the study due to an infection. Herpes virus-associated infections occurred in 5.7% of ublituximab recipients and in 4.6% of teriflunomide recipients. All were grade 1 or 2 in severity and resolved. No opportunistic infections or cases of progressive multifocal leukoencephalopathy (PML) were reported in either treatment group over the 96-week study period; however, this observation must be interpreted with caution given the limited duration of follow-up and the need for longer-term surveillance.

### Hypogammaglobulinemia and decreased leukocyte count

In the ULTIMATE I and II RCTs, hypogammaglobulinemia primarily affected immunoglobulin (Ig) M levels [15]. At Week 96, a greater proportion of participants treated with ublituximab had IgM levels below the lower limit of normal (LLN) (20.9%) compared to those receiving teriflunomide (4.9%). IgG levels were below the LLN in 6.5% of ublituximab-treated patients and 4.9% of teriflunomide-treated patients. These patterns are consistent with a class effect of anti-CD20 therapies [3, 4, 6, 8, 9, 11] and support the need for periodic immunoglobulin monitoring during long-term treatment. In contrast, IgA levels were similarly affected in both treatment groups, with 2.4% of ublituximab-treated patients and 2.0% of teriflunomide-treated patients having IgA levels below the LLN. Leukopenia was reported in a small proportion of RMS patients, occurring in 2.0% of those treated with ublituximab and 3.5% of those treated with teriflunomide. Lymphopenia was more frequently observed in the ublituximab group (9.7%) compared to the teriflunomide group (1.1%). In contrast, neutropenia was slightly less common in ublituximab-treated participants (3.3%) than in those receiving teriflunomide (4.2%).

### Immunogenicity and antibodies against ublituximab

During the ULTIMATE I and II RCTs, serum samples were analyzed for anti-drug antibodies (ADAs) and neutralizing antibodies (NAbs) over the 96-week treatment period. Among the 543 RMS patients receiving ublituximab, 17.8% tested positive for ADAs at baseline, while 86.5% tested positive at least once during the study [34]. The prevalence of NAbs was lower, with 2.4% of patients testing positive at baseline and 6.4% testing positive at least once post-baseline. The development of treatment-emergent ADAs and NAbs peaked at Week 24 and subsequently declined. Although ADA prevalence in ublituximab-treated patients was comparable to that observed with rituximab (reported range: 7.0–28.6%) [17, 35, 36], it was higher than what has been observed with other anti-CD20 monoclonal antibodies, including ocrelizumab (0.4–1.9%) [37, 38] and ofatumumab (0.2%) [39]. However, the higher ADA rates with ublituximab may reflect differences in detection methods, as ADAs in ublituximab-treated patients were quantified using a drug-tolerant electrochemiluminescent assay, whereas other studies employed less sensitive radioimmunoassays. Importantly, the presence of ADAs did not affect B-cell depletion, efficacy (e.g., impact on ARR or the number of new or enlarging T2-hyperintense WM lesions) as well as the incidence of IRRs [7, 24, 34].

### Neoplasms

No cases of neoplasms were reported in the phase II RCT [22]. In the Phase III RCTs, the incidence of neoplasms remained low. In the ublituximab group, two patients (0.4%) developed neoplasms, including one case of endometrial cancer and one case of uterine cancer [15]. In the teriflunomide group, one patient (0.2%) developed a tongue neoplasm [15]. There was no evidence of an increased risk of malignancy associated with ublituximab during the study period.

## Long-term efficacy and safety: the 5-year open-label extension

Following the 96-week double-blind phase, 85.4% of RMS patients in the ublituximab group (*n* = 422) and 87.4% of those in the teriflunomide group (*n* = 429) transitioned into the open-label extension (OLE) phase, where they either continued treatment with ublituximab or switched from teriflunomide to ublituximab [40]. While these 5-year data are valuable, their interpretation requires caution given the open-label design.

### Efficacy

RMS patients who continued ublituximab throughout the OLE maintained consistently low ARR values throughout follow-up, with levels remaining at 0.053 in Year 3, 0.032 in Year 4, and 0.020 in Year 5 [40]. For RMS patients who switched from teriflunomide to ublituximab, the ARR showed a 58.4% relative reduction during the first year of the OLE, from 0.182 to 0.076 (*p* < 0.0001), then continuing to decline and reaching 0.045 by Year 5. Although ARR remained slightly higher in the switch group compared to the continuous ublituximab group, this difference did not reach statistical significance at any timepoint (*p* ≥ 0.0727). The five-year analysis also showed a significant relative reduction in the risk of 24-week CDP among patients who remained on ublituximab, compared to those who switched from teriflunomide. Specifically, the risk of 24-week CDP was reduced by 38.8% in the continuous ublituximab group ([HR] = 0.612, 95% CI = 0.414–0.904, *p* = 0.0126). By Year 5, 8.0% of patients in the continuous ublituximab group experienced 24-week CDP, compared to 14.3% of those who switched from teriflunomide to ublituximab (*p* = 0.0032). Even though these results are derived from small absolute event numbers and therefore require cautious interpretation, they suggest that earlier initiation of ublituximab may have beneficial effects in limiting disability progression when evaluated over longer follow-up periods. RMS patients who remained on ublituximab were 47.2% more likely to achieve CDI than those who switched from teriflunomide (HR = 1.472, 95% CI = 1.048; 2.067, *p* = 0.0249). By Year 5, CDI was observed in 17.0% of RMS patients in the continuous ublituximab group and 12.2% of patients in the group that switched from teriflunomide to ublituximab (*p* = 0.0382).

### Safety and tolerability

The rate of treatment-emergent AEs in the pooled ublituximab cohort was 205.08 events per 100 patient-years, which was significantly lower than the 374.84 events per 100 patient-years observed during the double-blind phase [40]. The incidence of SAEs remained stable, occurring at a rate of 5.88 events per 100 patient-years in the pooled ublituximab OLE cohort, comparable to the rate observed during the double-blind phase (5.59 events per 100 patient-years) [40]. Treatment discontinuations due to AEs were rare, with rates of 1.69 events per 100 patient-years in the pooled ublituximab cohort and 1.66 events per 100 patient-years during the double-blind phase.

The overall infection rate in the pooled ublituximab cohort was 48.61 events per 100 patient-years, which was significantly lower than the 80.92 events per 100 patient-years recorded during the double-blind phase. Serious infections remained infrequent, occurring at a rate of 2.58 events per 100 patient-years in the pooled ublituximab OLE cohort, similar to the 2.10 events per 100 patient-years observed in the double-blind phase. Importantly, no cases of PML were reported in any RMS patients receiving ublituximab as of the data cutoff in January 1, 2024.

Even though these lower rates of AEs and infections may partly reflect survivor bias, as patients who tolerated therapy well and responded to treatment were more likely to continue into the OLE, they also suggest a good safety profile over time.

IRRs continued to decrease in frequency over time. In the pooled ublituximab cohort, IRRs occurred at a rate of 26.69 events per 100 patient-years, a marked reduction compared to the 54.12 events per 100 patient-years reported in the double-blind phase.

The incidence of malignancies remained extremely low, with a rate of 0.17 events per 100 patient-years, consistent across both the double-blind phase and the pooled ublituximab OLE cohort.

Serum Ig levels remained stable throughout the five-year treatment period. In the continuous ublituximab cohort, mean IgM levels were 0.69 g/L, remaining above the lower limit of normal (0.4 g/L), while mean IgG levels were 8.06 g/L, above the lower limit of normal (5.65 g/L). No correlation was observed between reductions in Ig levels and the incidence of infections, further supporting the long-term safety profile of ublituximab. Nonetheless, with prolonged B-cell depletion the cumulative risk of hypogammaglobulinemia may increase; therefore, ongoing immunoglobulin monitoring remains warranted during long-term therapy.

### THE ENHANCE study

The ENHANCE study (NCT05877963) is an open-label, multi-center, 48-week trial designed to evaluate the safety, efficacy, and tolerability of transitioning RMS patients from other DMTs to ublituximab [41]. The study aims to explore two optimized dosing regimens: (1) eliminating the initial 150 mg dose for RMS patients who are already B-cell depleted and (2) shortening infusion durations for the 450 mg full dose to 45 or 30 min.

Eligible participants were adults aged 18–65 years with a diagnosis of RMS according to the 2017 revised McDonald criteria, an EDSS score of ≤ 5.5, and prior treatment with a DMT. Initially, only B-cell depleted RMS patients transitioning from an anti-CD20 therapy (ocrelizumab, rituximab, or ofatumumab) were included. However, a protocol amendment later expanded eligibility to include non-B-cell depleted RMS patients transitioning from other DMTs, such as natalizumab, dimethyl fumarate, fingolimod, teriflunomide, and interferon beta. B-cell depletion was defined as a baseline B-cell count of < 10 cells/µL.

The currently available findings are preliminary and indicate that 126 RMS patients were enrolled, including 86 B-cell depleted and 40 non-depleted RMS patients. Among the B-cell depleted RMS patients, 94% had previously been treated with ocrelizumab and 6% with ofatumumab. In the non-depleted group, the most common prior DMTs were natalizumab (28%), dimethyl fumarate (15%), ocrelizumab (12%), and fingolimod (12%).

The primary focus of the ENHANCE study was the safety and tolerability of optimized ublituximab dosing regimens. On Day 1, 100% of initial ublituximab infusions (both 150 mg and 450 mg doses) were successfully completed. The infusion duration or dose did not affect the completion rate or the incidence of infusion modifications. A total of 16% of B-cell-depleted patients (*n* = 14) experienced an IRR after the Day 1 dose, whereas 8% of non-depleted RMS patients (*n* = 3) reported an IRR. Notably, all RMS patients received an antipyretic before each infusion. Although these IRR rates may appear numerically lower than those reported in the pivotal ULTIMATE RCTs, no direct comparison between the two studies should be made, due to the small sample size of ENHANCE and differences in study design. The most commonly reported Grade 1 IRR symptoms were throat irritation (*n* = 7) and headache (*n* = 5). A single Grade 2 IRR (minor throat itchiness) resolved without requiring modification to the infusion protocol. By Week 24, all 450 mg ublituximab infusions were completed successfully and well tolerated at the standard 60-min infusion duration (*n* = 13), as well as at shorter infusion durations of 45 min (*n* = 13) and 30 min (*n* = 12). A total of 82% of RMS patients received a non-drowsy antihistamine before their Week 24 infusion. Only three RMS patients experienced Grade 1 IRRs, including itching, throat irritation, and headache. All IRRs were mild (grade 1) and fully resolved. The recruitment of additional patients to increase sample size is ongoing.

These interim data suggest that ublituximab may be safely administered using an optimized dosing strategy, including shorter infusion times and elimination of the initial 150 mg dose in B-cell depleted patients, without compromising safety or tolerability. However, these results should be interpreted with caution given the small sample size and preliminary nature of the data.

## Experts’ perspective on ublituximab use for RMS patients

From an expert perspective, ublituximab is a recently approved anti-CD20 monoclonal antibody that broadens treatment options for RMS. When comparing ublituximab with other approved anti-CD20 monoclonal antibodies, such as ocrelizumab and ofatumumab, its efficacy in reducing relapse rates and MRI activity appears broadly consistent with this class, based on indirect evidence from pivotal trials. Likewise, its safety profile aligns with class-related observations, including risks of infusion-related reactions, infections, and potential hypogammaglobulinemia, although longer-term real-world data are needed to confirm these similarities. However, its structural and functional enhancements may distinguish it from other anti-CD20 therapies, making it a compelling choice for certain patient populations. As a glycoengineered chimeric IgG1 mAb, ublituximab has an Fc region optimized to enhance affinity for FcγRIIIa receptors on natural killer cells. This modification significantly improves ADCC, leading to a more efficient depletion of CD20-positive B-cells. Importantly, ublituximab maintains CDC, ensuring a broader mechanism of action that enhances its ability to suppress B-cell-mediated inflammation. These mechanistic features are theoretical and their precise clinical implications remain to be confirmed through dedicated studies.

One of the key advantages of ublituximab is its rapidity in depleting B-cells. Phase III RCTs showed a 96% reduction in CD19-positive B-cells within 24 h of the first infusion. While further research is needed to assess the clinical implications of such rapid depletion, this early reduction in B-cells may provide an immediate anti-inflammatory effect, potentially leading to quicker disease stabilization and earlier suppression of new MS activity.

Furthermore, the efficient infusion protocol of ublituximab, with subsequent maintenance doses requiring only 1 h twice a year, offers substantial advantages in terms of patient convenience and healthcare system efficiency. The shorter infusion times reduce burden on infusion centers and associated healthcare costs, improving accessibility for patients requiring long-term treatment.

Regarding the efficacy, the ULTIMATE I and II RCTs consistently showed that ublituximab significantly reduces disease activity in RMS. The 59% and 49% relative reductions in ARR compared to teriflunomide highlight its strong clinical efficacy. Additionally, the near-complete suppression of Gd-enhancing lesions and new/enlarging T2-hyperintense WM lesions reinforces its potent anti-inflammatory effect. This is further confirmed by the high proportion of RMS patients achieving NEDA-3, with 44.6% and 43.0% of patients in ULTIMATE I and II, respectively, substantially outperforming teriflunomide.

Given its rapid action, efficient administration, and strong efficacy, ublituximab may be considered for treatment-naïve patients with newly diagnosed RMS and high disease activity that may benefit from immediate and robust inflammatory suppression, leading to better early disease control. Moreover, RMS patients inadequately controlled on injectable or oral DMTs can benefit from superior suppression of inflammatory activity and biannual dosing, which reduces treatment burden compared to daily or more frequent dosing regimens. Additionally, ublituximab could be considered as a potential option for patients transitioning from natalizumab, particularly those at risk of PML, although this remains speculative and requires further supporting evidence. Similarly, the four-month washout period may be advantageous for women planning pregnancy, as reported in Summary of Product Characteristics (SmPC). However, its safety during pregnancy and lactation has not been established and must be confirmed in future studies. Evidence from ocrelizumab and ofatumumab suggests that anti-CD20 therapies can generally be managed safely around conception and pregnancy when planned appropriately. Particular caution is warranted in elderly patients, those with a history of malignancy, or individuals with increased infection susceptibility, as these populations may be at higher risk of adverse events. Careful patient selection and long-term real-world data are essential to clarify the safety and risk–benefit profile of ublituximab in these groups.

Despite its strong efficacy profile, certain aspects warrant further investigation. In ULTIMATE I and II, ublituximab did not show a significant difference in CDP or brain atrophy relative reduction compared to teriflunomide. This raises questions regarding its potential neuroprotective effects, particularly in progressive MS patients or those at higher risk of long-term disability accumulation. The established efficacy of ocrelizumab in primary progressive MS provides an important benchmark when considering the potential role of ublituximab in this population. However, as no data are currently available for ublituximab in progressive forms of MS, such considerations remain speculative and should be the focus of future research. However, it should be also noted that teriflunomide is an active comparator that has well-documented neuroprotective properties, including effects on disability progression and brain volume preservation [42–45], which may have influenced the comparative results. In line with this, the number of patients who experienced 24-week CDP was very small in both treatment groups (3.3% versus 4.8%). However, longer follow-up is essential to fully elucidate the impact of ublituximab on neurodegeneration and irreversible disability progression. Supporting this hypothesis, at the 5-year follow-up, a significant difference in the proportion of patients with CDP was observed: 8.0% in the continuous ublituximab group versus 14.3% in those who switched from teriflunomide to ublituximab, suggesting a potential benefit of earlier initiation. Given that inflammatory activity and new WM lesion formation can influence disability progression over a two- to three-year period [46], the full neuroprotective potential of ublituximab may take longer to become evident. Furthermore, early evaluations of brain volume loss may be influenced by pseudo-atrophy [47], a well-recognized phenomenon in the months following the initiation of potent anti-inflammatory therapy. Pseudo-atrophy reflects the resolution of edema and inflammation, rather than true neuro-axonal loss, complicating early assessments of neuroprotection. The possibility that ublituximab has neuroprotective effects is an intriguing but unproven hypothesis. Future long-term studies with appropriate endpoints such as progression independent or relapse activity (PIRA), MRI markers of neurodegeneration (e.g., spinal cord and gray matter atrophy) chronic inflammation (e.g., chronic active lesions), and body fluid biomarkers (e.g., neurofilament light chain and glial fibrillary acidic protein [GFAP] levels) are needed to better explore whether ublituximab truly impacts neurodegeneration and irreversible disability progression [48–50].

IRRs remain the most frequently reported adverse events associated with ublituximab, particularly during the first infusion. These reactions were generally mild to moderate, with common symptoms including fever, headache, nausea, and flushing. Importantly, the glycoengineered design of ublituximab is hypothesized to potentially reduce IRR rates by enhancing ADCC compared to less optimized anti-CD20 therapies. However, this remains speculative and must be confirmed in dedicated comparative studies. Premedication with corticosteroids and antihistamines effectively mitigated these reactions, and IRR incidence declined significantly with subsequent infusions. Accordingly, the impact of this AE seems limited and manageable in the clinical context.

Immunogenicity is another critical factor in evaluating the long-term safety and efficacy of mAb therapies. The development of ADAs and NAbs can potentially compromise therapeutic efficacy or increase hypersensitivity risks. In the ULTIMATE I and II RCTs, ADAs were detected in up to 86.5% of ublituximab-treated patients [34]. Importantly, these antibodies were not associated with negative effects on B-cell depletion, relapse control, MRI lesion suppression, or IRR incidence during the study period. Nevertheless, their long-term clinical significance remains uncertain, and continued monitoring in real-world studies is essential to determine whether ADA accumulation could impact treatment durability or safety. Long-term studies and real-world evidence will be essential in determining whether ADA development affects treatment durability in broader patient populations.

While the safety profile of ublituximab appears robust, long-term data are still needed to further assess the risks of hypogammaglobulinemia, infection susceptibility, and neoplasm development. Real-world data from larger, more diverse patient populations will be critical to understanding its long-term tolerability and potential risks.

## Data Availability

This is a review article. No original data were used.
